# What does the UK public want from academic science communication?

**DOI:** 10.12688/f1000research.8815.1

**Published:** 2016-06-07

**Authors:** James Redfern, Sam Illingworth, Joanna Verran

**Affiliations:** 1 School of Healthcare Science, Manchester Metropolitan University, Manchester, UK; 2 School of Research, Enterprise & Innovation, Manchester Metropolitan University, Manchester, UK

**Keywords:** Science communication, public engagement, outreach

## Abstract

The overall aim of public academic science communication is to engage a non-scientist with a particular field of science and/or research topic, often driven by the expertise of the academic. An e-survey was designed to provide insight into respondent’s current and future engagement with science communication activities. Respondents provided a wide range of ideas and concerns as to the ‘common practice’ of academic science communication, and whilst they support some of these popular approaches (such as open-door events and science festivals), there are alternatives that may enable wider engagement. Suggestions of internet-based approaches and digital media were strongly encouraged, and although respondents found merits in methods such as science festivals, limitations such as geography, time and topic of interest were a barrier to engagement for some. Academics and scientists need to think carefully about how they plan their science communication activities and carry out evaluations, including considering the point of view of the public, as although defaulting to hands-on open door events at their university may seem like the expected standard, it may not be the best way to reach the intended audience.

## Introduction

Engaging members of the lay public with science has become a business-as-usual activity for many scientists within academia. Although a widely accepted definition is yet to exist, science communication can be considered an umbrella term for public engagement, outreach, widening participation etc., with the specifics depending on the mode of delivery, aim, location, intended audience, and institutional preference ^1^. Common examples of science communication include university open door events, science festivals, school visits, writing for the popular press, providing engaging presentations in easily-accessible locations (pubs, cafes etc.), using social media, and producing digital media such as podcasts and video content. The overall aim in all cases is primarily to engage a non-scientist with a particular field of science and/or research topic, often driven by the expertise of the academic.

Support for these science communication activities continues to grow following a pivotal report by the Wellcome Trust and the Office of Science and Technology in the UK, which recommended moving away from a deficit model ^2^, whereby scientists assume the public have a lesser knowledge of science and provide information to fill any gaps. The deficit model has been discussed and criticized widely by many [for example 3– 5], with science communicators favouring a contextual approach to establish dialogue between the experts and the members of the general public. This approach has been incorporated into the ethos and policy of many leading public engagement organisations such as the National Coordinating Centre for Public Engagement (NCCPE), which defined public engagement as “…a two-way process, involving interaction and listening, with the goal of generating mutual benefit.” ^6^.

The UK Research Council (RCUK) representing all of the research councils in the UK, and many other research funders (e.g. The Wellcome Trust) require all applicants to integrate science communication in their research plan. Additionally, the 2014 Research Excellence Framework (REF) exercise, whereby the UK Government assessed all university research output in order to distribute funding, included an assessment of research impact, which was often supported with evidence of effective science communication, thereby helping to establish this “as a core part of business, not just good intentions” ^7^.

It is therefore no surprise that there is a growing trend within universities to encourage their academic staff to undertake activities that may be considered as science communication. Researchers have indicated many important motivations for these activities, including increasing the public’s interest of science and awareness as well as an appreciation of science and scientists ^8, 9^.

Science festivals often encompass obvious outputs of academic public engagement. Described as a ‘celebration’ of science, technology, engineering and related aspects, with the intention to engage non-specialists with a time-limited recurring frequency, they are growing in number globally, with the UK hosting more than any other country ^10^. 

Whilst the attraction and effect of science festivals on participating public are well researched [e.g. 11– 13], there is little in the literature to suggest that this is the preferred method of communication for the audience (‘the public’), nor how members of the public would actually like to engage with academic scientists if they had the choice. Science festivals are of course not unique in this context, with many different forms of communique from ‘popular science’ books ^14^ to blogs ^14, 15^ and podcasts ^15, 16^ often receiving support depending on the individual preferences of the author/presenter/scientist. Indeed, incidental evidence collected by the authors of this study suggest that the mode of delivery, methods, and most importantly the scientific topic of many science communication strategies are not decided in consultation with the general public, but are instead determined by constraints such as time, resources, skills and finance. This paper describes a preliminary small-scale study to assess what science communication output the public may like to see, and suggests how academics may be able to better engage with them as a result of this.

## Method

A questionnaire-based e-survey was designed with ten questions, four of which captured demographic details of the respondents ( Table 1). The aim of the survey was to provide insight into respondent’s current and future engagement with science communication and/or public engagement activities. The intended respondents were anybody who did not identify as a scientist, therefore the only exlusion critieria was self-identification as a scientist.

**Table 1.** List of survey questions.

Question number	Question
1	Where do you live? (Town and Country e.g., Manchester, UK)
2	**Gender? (E.g. male, female)**
3	Age?
4	What is your highest level of qualification? (if not from the UK please specify in ‘Other’ box)
5.1	Are you interested in science? (if you would like to expand on or explain this answer, please feel free to do so below)
5.2	Could you give us a little more detail e.g. what is it about science that you particularly like or dislike?
6	How often do you engage with science through the following: • TV • Radio • Newspapers • Magazines • Internet • Specific websites (e.g. blogs) • Non-specific websites (e.g. news) • Social media e.g. Twitter & Facebook • From a friend or relative • Museums • Science festivals and other science events • Other
7.1	Scientists and other organisations such as museums and universities may encourage people to attend events to see/discuss/participate with science. What would encourage you to visit a science festival/university open day/museum to engage with scientists?
7.2	Have you ever attended one of these events? If so, then what type of event(s) was it?
7.3	Would knowing what topic of science that was on display/being discussed influence your decision to attend?
8	How would you suggest scientists could engage with you more effectively? For example, TV, social media, online resources, podcasts, blogs, live events, hands-on/interactive displays, science open-door events, etc.
9	Would you rather science events were focused on children, teenagers and adults or split equally between all ages?
10	What is it about science that would make you interested in engaging with the subject more?

The survey was made available for one month (May 2015) and advertised through the social media accounts of the authors (Twitter, Facebook and LinkedIn), with participants encouraged to share the survey with anybody who did not identify as a scientist. Ethical clearance was obtained prior to data collection from the Manchester Metropolitan University Ethics Board (document number SE141518), and carried out according to the British Educational Research Association’s (BERA) ethical guidelines for educational research; all responses were collected anonymously. Data were analysed using NVivo ^®^ (v10; QSR International Pty Ltd, Melbourne), where individual responses were coded into themes that were dertermined after an initial analyisis for the purpose of detailed analysis and discussion. 

## Respondents

A total of 112 responses were collected from respondents from ten countries. The nationalities of the participants were: United Kingdom (71.4%), United States of America (17%), Canada (2.7%), Netherlands (1.8%), Australia (1.8%), Germany (0.9%), Tanzania (0.9%), New Zealand (0.9%), Norway (0.9%), Spain (0.9%) with 0.9% not providing an answer. There were more male responses (56.3%) than female (42.9%) with 0.9% not providing an answer.

The most frequent (34.8%) age of respondents was between 20–29 years old, followed by 30–39 years old (20.5%), 50–59 years old (18.8%), 40–49 years old (12.5%), <19 years old (6.3%), 60+ years old (6.3%) and 0.9% not providing an answer. Respondents reported a wide range of educational backgrounds from no formal qualifications to doctoral degrees ( Table 2). All respondents replied no when asked if they were a scientist.

**Table 2.** Percentage of responses provided to the question "What is your highest level of qualification?" n=112.

Qualification	Percentage
No formal qualification	0.9
1–4 GCSEs	4.5
5+ GCSEs	1.8
Apprenticeships	6.3
A Levels	15.2
Undergraduate degree	42.9
Masters degree	18.8
Doctoral degree	2.7
Non-UK (equivalent to A Level)	0.9
Non-UK (equivalent to Undergraduate degree)	0.9
Non-UK (equivalent to Masters degree)	1.8
Non-UK (equivalent to high school education)	3.6

## Results and discussion

Survey responses Click here for additional data file. Copyright: © 2016 Redfern J et al. 2016 Data associated with the article are available under the terms of the Creative Commons Zero "No rights reserved" data waiver (CC0 1.0 Public domain dedication).

### Current views and science communication

**Figure 1.**
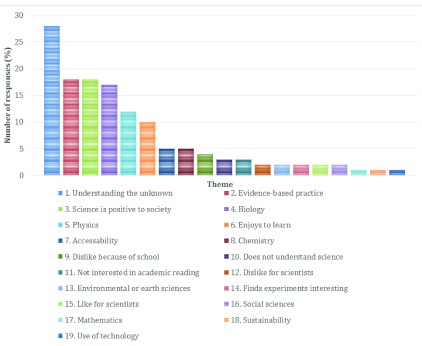
Responses categorised into themes relating to respondents current interest in science.

The majority of respondents agreed that they were interested in science (87.5%), with only 7.1% stating the opposite. Of the remaining respondents, 4.5% selected ‘other’ and provided mixed opinions whilst 0.9% did not provide an answer. Respondents provided a range of comments, which were coded into 18 different themes ( Figure 1). The most common reason (21%) given for interest in science attempts to understand the unknown.

*“It's based on facts, discoveries and inventions improve our lives, it's interesting to know how the world works”*

The second and third most common themes were the ethos of science (scientific method and its basis in evidence-based practice), and the perception that science is beneficial to society (both 13%). One respondent had mixed views about these aspects, noting that whilst science allows people to discover the unknown, the ‘dogma’ of science and the inability to access scientific literature made it difficult for people to interact with. This is interesting and supportive to the ongoing Open Access movement within academia, attempting to make all peer-reviewed research manuscripts freely available to everyone. Other respondents specified favoured disciplines; biology (12.5%), physics (8.8%), chemistry (3.7%), environmental or earth sciences (1.5%), social sciences (1.5%), mathematics (0.7%) and sustainability studies (0.7%).

Of the respondents who stated that they had no interest in science, no explanation related to a specific scientific discipline. Instead they referred to personal experience with science education (50%), the conflict scientific method can have with religion (25%), or a personal dislike of scientists themselves (25%).

*“Snobs, know it all! Better than others just because they are intelligent, boring and thinking everyone should know what they know!”*

One respondent, when discussing their education, detailed how intrigued they often are, but are made to feel inadequate and lacking in capacity and understanding.

*“…like it’s no big deal, and my wonder and awe is ill inspired”.*

**Figure 2.**
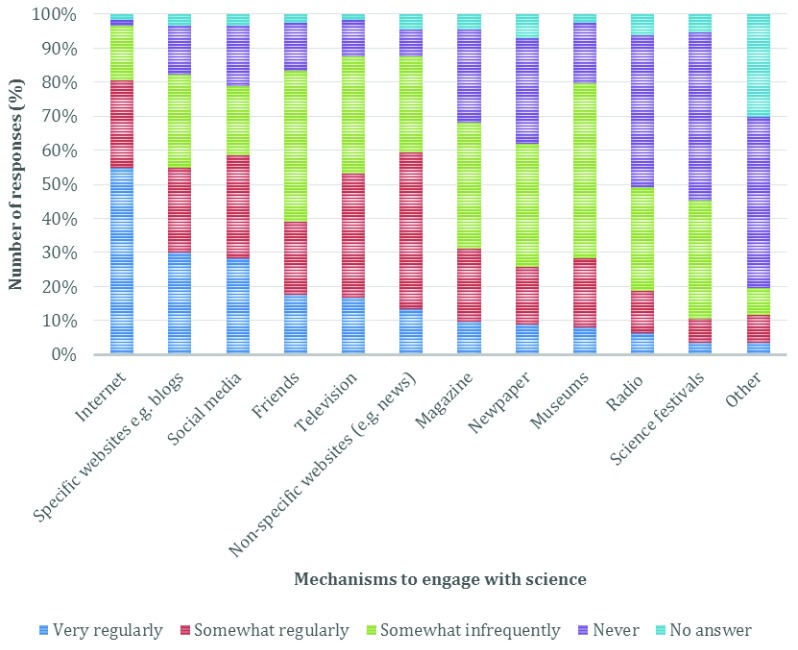
Distribution of answers regarding how often respondents use different mechanisms to interact with scientific information (displayed as percentage of overall respondents).

To understand how respondents engaged with science in their day-to-day lives, they were asked to select either very regularly, somewhat regularly, somewhat infrequently or never ( Figure 2). When asked about different engagement mechanisms, the most frequently selected mechanism was via the Internet, with 55% of respondents using it very regularly, and a further 25% somewhat regularly. A further four mechanisms were used by over 50% of respondents at least somewhat regularly, these were: non-specific websites such as news pages, social media (28% very regular, 30% somewhat regular), specific websites such as blogs (30% very regularly, 24% somewhat regularly), and television (16.8% very regularly, 36.2% somewhat regularly). Interestingly, the top four are all online methods of communication, and represent all of the online options provided. Academics too are actively engaged with social media to network, discuss, plan and carryout studies ^17^, with 80–90% of research scientists being at least aware of Twitter and Facebook ^18^. This rich and existing community of academics could therefore be utilised more frequently and imaginatively to engage members of the public.

Traditional media, i.e. magazines (10% very regular and 21% somewhat regularly), newspapers (9% very regular and 17% somewhat regularly) and radio (6.25% very regular & 12.5% somewhat regularly) are used less often to access science. A small group of respondents used physical events, such as museum visits (8% very regularly and 20% somewhat regularly), or attendance at science festivals to engage (3.5% very regular and 7% somewhat regularly). As noted in the introduction, academics practising science communication and engagement appear to favour these types of activities for public engagement events ^19^, perhaps due to opportunity to adhere to a more standard format they are comfortable with – e.g. standing in front of people and explaining.

Some respondents noted other methods for engagement with science that were not included in the survey. They were: multimedia content (e.g. DVD and on-demand streaming services, n=3), amateur science (e.g. amateur astronomer club or designing experiments for their children, n=3), through their workplace/job (e.g. working in a library, n=2), and by reading (books and academic literature, n=2).

Respondents were also asked which events/activities aimed at engaging the public with science, if any, they had previously attended or participated in. Of all the respondents who provided a 33.8% of them had not attended any science engagement events.

*“No, I've never been aware of such an event.”*

Museums were the most popular attended location (23.5%), with 13.2% attending but not specifying details. Universities and science festivals/fairs had a similar response rate (11.8% and 10.3% respectively); only 1.5% had previously engaged in a science communication activity online. This contrasts with the responses from the previous question, which revealed that the Internet was used to actively engage with science ( Figure 2). It seems that if the curiosity is driven by the consumer rather than prescribed by the academic, then engagement with science is better recognised. The role of the scientist in creating activities is of little concern.

### How can we better engage with the public?

**Figure 3.**
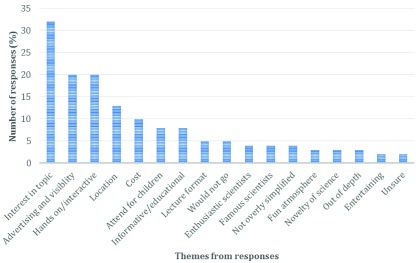
Distribution of answers regarding what would encourage respondents to attend a science event at a university or museum.

The following questions asked respondents how scientists and other organisations such as museums and universities may encourage people to attend events to see/discuss/participate with science. Respondents provided a wide range of reasons (n=146) or actions that may alter their decision to attend a science communication/outreach event ( Figure 3). The most common theme related to their interest in the topic (21.9%). Suggested topics were wide-ranging, from science of practical value to the respondent, to any topic that the respondent may find interesting as long as it followed scientific methodology.

*“Novel exhibitions (e.g. opportunity to try a jet pack)”*

Advertising and visibility of events were also considered (13.7%), with many respondents suggesting that they rarely see science communication events being advertised, but generally would attend if they knew far enough in advance. Some respondents elaborated on this, suggesting that advertising should be carried out on social media (n=6) whilst two respondents suggested that advertising be written with inviting language, so as not to intimidate or patronise any potential attendees.

Also of concern was the ability to engage with a hands-on practical demonstration with “more than just storyboards” (13.7%) as well as the geographic location (8.9%) of the activities, as some respondents lived in locations away from major universities or other organisations and so did not have the option to attend without travelling. These responses provide an interesting consideration for scientists; whilst many may consider hands-on practical demonstrations of science to be a valuable tool for the effective communication of science (again, similar to experiences they may have within the undergraduate classroom), participation will be limited to those within the commutable area. Providing further support to develop science communication activities within the digital landscape may be one way to enable participation with people in any geographical location, and is a change that is likely to be embraced given previous answers discussed relating to Internet-based engagement ( Figure 2). 

Despite the interesting opportunities for active involvement that citizen science and crowd sourcing can offer [e.g. 20], only two respondents specified they would like experiments they could investigate themselves. Interestingly, the lecture format was suggested to a lesser extent (3.4%), a low approval for an anecdotally common format. Other considerations included cost (notably the requirement for events to be free, 6.8%) and the inclusion of enthusiastic (2.7%), or famous (2.7%) scientists.

**Figure 4.**
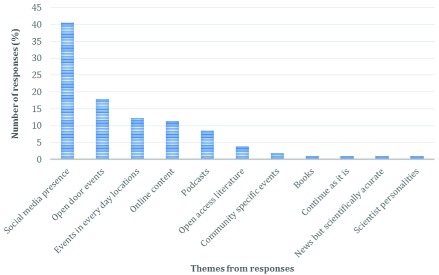
Distribution of answers regarding what the respondent suggested scientists could do to engage better with them. n=106.

**Figure 5.**
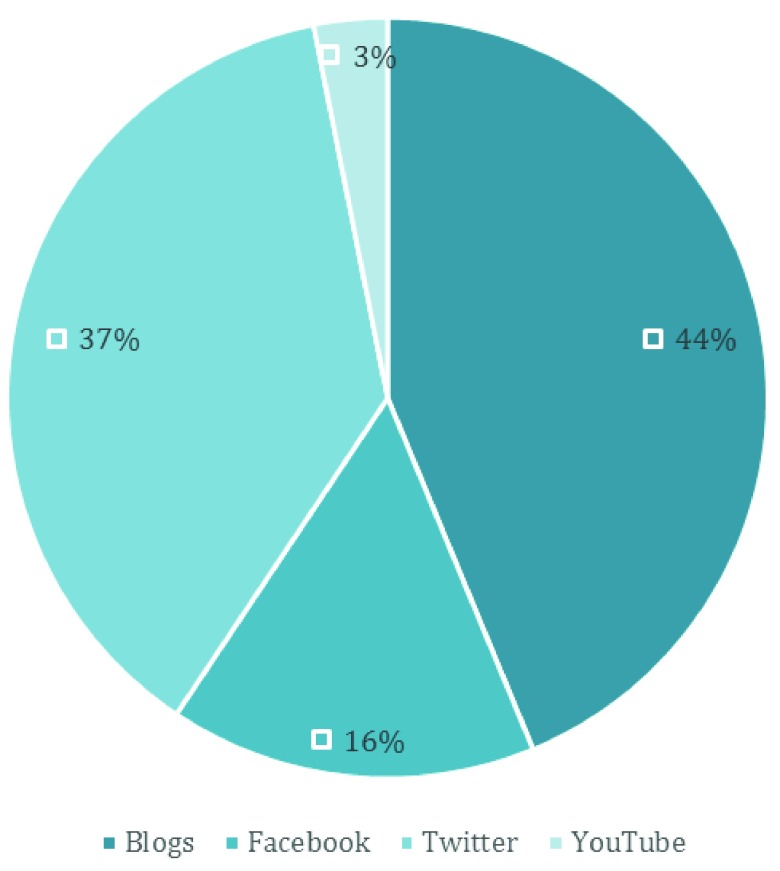
Distribution of particular elements of social media that respondents described if they selected social media as an answer when considering how scientists can better engage with them ( Figure 4). Not all respondents who selected social media provided a specific answer. n=32.

Respondents were asked to consider how they themselves could be better engaged by scientists trying to communicate scientific information ( Figure 4). The most common answer was to bring scientific information to social media (41%). Of these respondents, 34% specified a type of social media ( Figure 5), the most common being blogs, followed by Twitter, Facebook and YouTube.

Suggestions for output content via social media were, interestingly varied. The need for “idiot proof websites” and short scientific “facts” to gain people’s attention, contrasted with suggestions for “more detailed” and “accurate” online dialogue, reflecting the two types of social media suggested, Twitter, where currently only short snippets of information can be posted at any given time due to 180 character limits; and blogs, where the norm is to create pieces of writing of variable length, allowing more detail. Within the comments regarding social media, there was an overall consensus that its convenient nature makes it an attractive mode for scientists and audiences to interact.

The second most common theme was open-door events, but only one respondent provided any detailed thought, suggesting open-door events should take place within a University. Two respondents linked hands-on activities with open-door events, but it may be assumed from previous responses that people associate hands-on activities with open-door events, and thus did not feel the need to discuss them separately. Events in ‘real life’ locations (outside of typical engagement locations e.g. university or museum) were also suggested (12.2%). Including Café Scientifique ( http://cafescientifique.org) or The Green Man music festival ( http://www.greenman.net/).

Respondents also suggested that online resources or websites were useful (but not specifically social media, 11.3%). A slightly smaller percentage of respondents recommended podcasting as a medium to better engage them with science (8.5%). Debate, discussion and interviews were all mentioned with reference to podcasts. Other themes resulting from this question included providing open-access literature (3.8%), community-specific events (such as local astronomy clubs, 1.9%), non-fiction books on scientific topics, better news coverage of scientific topics and more famous scientist personalities (0.9%). Also, 0.9% of responses suggest scientists should continue as is, as they believed scientists were successfully achieving their aim in effectively communicating with the public.

The age at which engagement events should be aimed was also investigated. The majority of responses suggested events should be split equally between adult-oriented and child-oriented (60.2%). The number of respondents suggesting that events should be aimed at children, adults or teenagers alone were similar (12.5%, 9.8% and 9.8% respectively). A small number of respondents believed events should be designed for families as a whole (3.6%), whilst the same number felt that the level of pre-existing knowledge should be the definition of who an event is aimed at, not the person’s age.

## Conclusions

This study has provided new insights into how populations within the ‘general public’ may wish to engage with academic scientists who are interested and/or required to undertake elements of science communication. Whilst the authors note a variation in responses, that is, no single answer, the results highlight the complexity of communicating with the public which academic scientists need to understand further to ensure outcomes are as effective as possible.

The potential use of the Internet and digital technologies seems favoured by respondents, with less of a focus on more ‘traditional’ academic science communication activities such as science festivals and open door events. However, the public still want access to these types of events, with considerations such as time, cost, geographical location, clear/accessible advertising (e.g. through social media) and a non-patronising approach should be undertaken. Another key point is that of targeting age groups, with the majority of respondents keen to see adults engaged just as much as children. The type of information the public believe they would be interested in varies, however the themes of understanding the unknown, and the benefits science brings to society were noticeably popular. In order for academic science communicators to undertake activities of this nature, training needs to be introduced/amended to include the point of view of the audience, considering other more accessible methods such as online delivery. This will inevitably not be true for all activities, and even where appropriate, new skills, technology and advertising strategies are likely to be required.

The outcomes of this paper should pose as food-for-thought for proponent science communicators, with more research needed to be done to better understand how different groups of the wider community can be better served by science communication activities presented in different formats. What is evident is the need to think carefully about how scientists plan their science communication activities and carry out evaluations, including considering the point of view of the public, as although defaulting to hands-on open door events at their university may seem like the expected standard, it may not be the best way to reach the intended audience.

## Data availability

The data referenced by this article are under copyright with the following copyright statement: Copyright: © 2016 Redfern J et al.

Data associated with the article are available under the terms of the Creative Commons Zero "No rights reserved" data waiver (CC0 1.0 Public domain dedication). 

*F1000Research*: Dataset 1. Survey responses, 10.5256/f1000research.8815.d123881 ^21^
