# Supplementary material for: What does the UK public want from academic science communication?
Source: F1000Res. 2016 Jun 7;5:1261. [Version 1] doi: 10.12688/f1000research.8815.1 (PMC4916990; doi:10.12688/f1000research.8815.1)
Supplement: Peer review history

# Peer review: Referee response for version 1

**DOI:** 10.5256/f1000research.9490.r14240

**Published:** 2016-06-24

**Authors:** Helen L. Brown

**Affiliations:** 1 Department of Biomedical Sciences, Cardiff Metropolitan University, Cardiff, UK

This paper analyses the results of a questionnaire completed by a group of non-scientists. The questions focused on how the participants currently engage with science and how they might like to do so in the future. Overall the paper provides a well written description of the study and highlights the key results. This paper will be of particular interest to those within the science community who are heavily engaged in promoting and/or carrying out pubic engagement events as it highlights that the current model of outreach which is favoured by many providers does not overlap with those activities favoured by participants in this study. This highlights that when designing events in order to engage and reach the widest possible audience the public should also be consulted.

Minor comments

**Introduction**

- Science festivals are discussed in the introduction but there is little description of the other outreach methods/events that were mentioned within the questionnaire. It might be of use to also give examples of some of the other engagement methods.

**Methods**

- it would be useful to apply some statistical analysis to the results, although I appreciate that statistical analysis may be limited by the sample size of the study.

Major comments

**Method**

- The paper analyses a questionnaire which was distributed via the authors social media accounts. This method of distribution may significantly bias the results obtained. In particular I would expect respondents who had answered a questionnaire advertised in the digital environment to encourage more online engagement from scientists, which was found. This should be considered when discussing the results.
- Similarly, although the authors refer to respondents as representing the 'general public' it could be argued that the respondents already have a enhanced level of scientific engagement, since they engage with the authors social media and chose to compete the questionnaire. This bias should also be addressed by the authors.

Overall I think this is a worthwhile and thought provoking preliminary study and hope that the authors will continue the work in the future. I would be very interested to see if the same conclusions can be drawn from a wider participant group and to look in more detail about what engagement methods the public find the most worthwhile.

I have read this submission. I believe that I have an appropriate level of expertise to confirm that it is of an acceptable scientific standard.

# Peer review: Referee response for version 1

**DOI:** 10.5256/f1000research.9490.r14242

**Published:** 2016-06-20

**Authors:** Massimo Caine

**Affiliations:** 1 Microbiology Unit, Department of Botany and Plant Biology, University of Geneva, Geneva, Switzerland

This manuscript develops an interesting and very much debated aspect of science communication *i.e.* which means ought be adopted by scientists and science communicators in order to successfully reach the lay-audience. Given the steep rise of different sources of scientific information (inside and outside the academic community), it is a timely submission that provides an analysis of the different possible ways to reduce the distance between scientific researchers and broad public. Importantly, this publication offers valuable food-for-thought coming from a privileged point of view, the layperson, attempting to address the flaws that the deficit model in communication may pose. As a matter of fact, by using an *ad-hoc* survey, Redfern *et al*. investigate which are the expectations of a non-scientific audience in terms of their engagement in scientific discussions. Remarkably, even though science fairs, festivals, citizen projects are the mainstream in many academic communication policies, the output of the survey intriguingly indicates that, if requested to choose, the public would prefer to be engaged with “time-saver” and less constrained approach like what can be found within the online environment.

 The authors have widely addressed the survey results, providing a perspective that should be carefully considered by academic policies, not only in terms of communication departments, but also in terms of scientists training. However, there are some minor points that, as a reader, I would be interested to see expanded and that the authors may wish to include in their final version:

1. the authors mention that science festivals that are more present in UK than in every other country - a brief description of some of them in terms of format and public-deliverables would be an asset for the introduction;
2. in order to better contextualize the preference of being engaged on an online format rather than with a science festival (fairs, open doors, hands-on workshops, citizen projects), it would be useful to know how much the selected audience of the survey have had the possibility to be “immunized” with such kind of events. Specifically, is there any correlation between the geographical origin of the public reached by the authors and the presence (or not) of science festival within the region? This would allow the authors to define weather if the lack of interest in science fairs is due to geographical proximity ( *i.e. *among the 112 selected participants, none or few of them lives close to the place where a festival has been taking place lately) or if it is an opinion generated by the comparison between the two way of engagement (festivals vs. online tools). In my opinion, this would clarify if festivals were not a preferred choice due to their non-commutable distance from the surveyed respondent.
3. Provided that one of the preferred choices of engagement are website and blogs, a brief description of some of these available supports and the way they deliver the scientific information would be a plus 

To conclude, I believe that, with this submission, Redfern *et al. *provide a seminal data-driven publication about the expectations that the broad public may have when being engaged with science. Even though, the sampling of the survey is made on a reduced scale (112 participants), it definitely poses the basis for further research which will push forward the methods used by the science communication community in order better reach the lay-audience in the fast-pace-changing and hyper-connected era that we are living in.

 I am looking forward to reading the final version of the manuscript and, in the meantime, I definitely recommend this article for being indexed.

I have read this submission. I believe that I have an appropriate level of expertise to confirm that it is of an acceptable scientific standard.

# Peer review: Referee response for version 1

**DOI:** 10.5256/f1000research.9490.r14241

**Published:** 2016-06-17

**Authors:** Laura Bowater

**Affiliations:** 1 Norwich Medical School, University of East Anglia, Norwich, UK

This paper does indeed provide food-for-thought for proponent science communicators. There are a plethora of science communication definitions and modes of delivery and this paper raises the intriguing possibility that science communicators may be getting left behind as the online revolution continues to take place and evolve. Although I feel there are a couple of caveats, for example with the audience reached through the particular sampling technique employed by the authors, this raises intriguing questions about the move towards two-way engagement and the difficulties that this will raise if online or indirect modes of delivery appear to be  most valued by the public or at least members of the public reached by these authors.

 I wonder if the authors would like to contextualise why they have chosen to talk about science festivals within the Introduction? It may also be of value to expand this section a tad. For example, authors could provide examples of science festivals within the UK and perhaps more globally. What does a typical science festival look like? How long does it last? Where does it take place? Who is invited?.. etc Also, perhaps the authors could describe why scientists choose this direct interaction as a medium? What are the advantages or disadvantages and is it a format that engenders two-way participatory engagement?

 Also, as the results indicate a desire for indirect science communication methods through online material etc perhaps the authors could expand a little on these types of delivery in the introduction too.

 In the introduction the authors state that:

 "This paper describes a preliminary small-scale study to assess what science communication output the public may like to see, and suggests how academics may be able to better engage with them as a result of this."

 I would temper this slightly. I suggest to the authors that instead they state:

 "This paper describes a preliminary small-scale study to assess which science communication outputs the public may like to see, and suggests how academics may be able to better engage with them, within their own personal constraints, as a result of this.

 With the methods section:

 The questionnaire raises some interesting questions. Would the authors be prepared to share why they chose these particular questions? This may help others who may be considering undertaking similar research into other aspects of science communication.

 With the results section:

 The authors state:

 "The majority of respondents agreed that they were interested in science (87.5%), with only 7.1% stating the opposite." How does this compare to MORI surveys carried out every four years in the UK that asked similar questions?

https://www.ipsos-mori.com/researchpublications/researcharchive/3357/Public-Attitudes-to-Science-2014.aspx

 Figure 1 I suggest replacing theme with themes.

 The authors state that "One respondent had mixed views about these aspects, noting that whilst science allows people to discover the unknown, the ‘dogma’ of science and the inability to access scientific literature made it difficult for people to interact with", would they consider illustrating this with a quote from the material?

 The authors suggest that interestingly, the top four are all online methods of communication, and represent all of the online options provided. Academics too are actively engaged with social media to network, discuss, plan and carry out studies, with 80–90% of research scientists being at least aware of Twitter and Facebook. This rich and existing community of academics could therefore be utilised more frequently and imaginatively to engage members of the public. This is an interesting comment and I know a lot of scientists would be glad to hear this. However I suggest that the authors temper this by stating...'in this study". It is worth remembering that respondents to the survey were contacted via online/ social media and you may be getting the views of the online-literate members of society that might not reflect the wider non-social media society.

 I wonder about the concept hinted at in the introduction, that academics practising science communication and engagement appear to favour these types of activities for public engagement events. I think there are many scientists who are turning to online indirect methods of sci comm to share areas of personal and wider science interest. Do the authors know if anything has been written about this at all? As a keen online provider of science material and functioning within these type of communities I wonder if I am seeing the result of my own biases as I feel all scientists are equally engaged this way as the community is vibrant and active and perhaps masks a wider science community that doesn't engage in this way. I am also intrigued that if this appears to be a preferred way for non-scientists to engage with science what does that mean for two-way engagement?

 Finally "Despite the interesting opportunities for active involvement that citizen science and crowd sourcing can offer" As someone actively involved in a citizen science project myself, I am intrigued by this result. It is thought provoking and deserves further attention.

 There are some interesting finding within this publication especially within the context of the deficit/fully engaged models that have framed the science communication discipline and community. I recommend that is is indexed.

I have read this submission. I believe that I have an appropriate level of expertise to confirm that it is of an acceptable scientific standard.
